# Royal Devon and Exeter Hospital

**Published:** 1915-06-12

**Authors:** Samuel S. Cole

**Affiliations:** Secretary.


### THE EDITOR'S LETTER-BOX.

### Royal Devon and Exeter Hospital.

*To the Editor of* THE HOSPITAL.

SIR,—My attention has been called to an article appearing in your columns on May 8, containing certain information respecting the nursing department of this institution, and, whilst agreeing with certain of the points which you raise, exception must be taken to the remarks as to the nurses' accommodation and the advantages offered to our staff for the purpose of joining the Royal National Pension Fund for Nurses. The information which your correspondent has given in respect to these two items is hardly correct, and rather implies that the interior arrangements of this institution are somewhat old-fashioned. I shall be glad therefore if when your representative is again in this neighbourhood he, or she, will take an early opportunity of seeking up-to-date information respecting this department.

Regarding the Royal National Pension Fund, this hospital *does* offer advantages to its nursing staff with a view to encouraging them to join the fund.

I shall be glad therefore if you will kindly correct the information in an early issue of your paper.—Yours faithfully, SAMUEL S. COLE,

May 28, 1915. Secretary.

[We are glad to find that the Royal Devon and Exeter Hospital does offer advantages to its nursing staff members who wish to join the Royal National Pension Fund, and regret we were misinformed in this connection. As to the accommodation for the nurses, the article published in our issue of May 8 was written after our representative's visit to the hospital and incorporated what she saw and what she was told there. We presume from the Secretary's letter that since that visit was paid the accommodation has been improved, for this action was very desirable. It would be satisfactory to hear the last of a former arrangement, whereby a ward had been divided into rooms, with a dark passage between, on one side the (staff?) nurses sleeping, and on the other the resident medical staff. These arrangements prevailed less than two years ago, but they have now, we presume, been altered, as they should be.—ED. THE HOSPITAL.]

### The Seamen's Hospital.

